# Stimulation of dendritic cells by DAMPs in ALA-PDT treated SCC tumor cells

**DOI:** 10.18632/oncotarget.5975

**Published:** 2015-11-26

**Authors:** Xiaojie Wang, Jie Ji, Haiyan Zhang, Zhixia Fan, Linglin Zhang, Lei Shi, Feifan Zhou, Wei R. Chen, Hongwei Wang, Xiuli Wang

**Affiliations:** ^1^ Shanghai Skin Disease Hospital, Shanghai 200443, China; ^2^ Biophotonics Research Laboratory, Center for Interdisciplinary Biomedical Education and Research, University of Central Oklahoma, Edmond, OK 73034, USA; ^3^ Huadong Hospital, Fudan University, Shanghai 200040, China

**Keywords:** danger-associated molecular patterns, 5-aminolevulinic acid mediated photodynamic therapy, squamous cell carcinoma, dendritic cells, immune responses

## Abstract

Photodynamic therapy (PDT) not only kills tumor cells directly but also rapidly recruits and activates immune cells favoring the development of antitumor adaptive immunity. It is believed that Topical 5-aminolevulinic acid mediated photodynamic therapy (ALA-PDT) can induce anti-tumor immune responses through dangerous signals damage-associated molecular patterns (DAMPs). In this study, we investigated the effect of ALA-PDT induced DAMPs on immune cells. We focused on the stimulation of dendritic cells by major DAMPs, enhanced the expression of calreticulin (CRT), heat shock proteins 70 (HSP70), and high mobility group box 1 (HMGB1), either individually or in combination. We evaluated *in vitro* and *in vivo* expressions of DAMPs induced by ALA-PDT using immunohistochemistry, western blot, and ELISA in a squamous cell carcinoma (SCC) mouse model. The role of DAMPs in the maturation of DCs potentiated by ALA-PDT-treated tumor cells was detected by FACS and ELISA. Our results showed that ALA-PDT enhanced the expression of CRT, HSP70, and HMGB1. These induced DAMPs played an important part in activating DCs by PDT-treated tumor cells, including phenotypic maturation (increase of surface expression of MHC-II, CD80, and CD86) and functional maturation (enhanced capability to secrete IFN-γ and IL-12). Furthermore, injecting ALA-PDT-treated tumor cells into naïve mice resulted in complete protection against cancer cells of the same origin. Our findings indicate that ALA-PDT can increase DAMPs and enhance tumor immunogenicity, providing a promising strategy for inducing a systemic anticancer immune response.

## INTRODUCTION

Recently it has been discovered that some anticancer treatments, such as chemotherapy, radiotherapy, and photodynamic therapy (PDT), kill cancer cells in an immunogenic fashion, thereby secondarily stimulating the immune system [1–2]. They can induce a form of programmed cancer cell death called immunogenic cell death (ICD) [3]. ICD is apoptotic in nature of its execution is immunogenic since it is accompanied with emission of dangerous signals damage-associated molecular patterns (DAMPs) [2]. Thus, these treatments are capable of not only directly killing cancer cells via intrinsic cytotoxicity, but also converting dying cancer cells into an anticancer vaccine. Furthermore, they generate a specific immune response against residual cancer cells and metastatic cells [4].

ICD depends on danger-associated molecular patterns (DAMPs) that activate innate immune cells for the generation of specific antitumor immunity [4]. DAMPs are molecules normally confined within live cells in subcellular compartments such as nucleus, cytosol, or biological membranes. As DAMPs are exposed and secreted by stressed/damaged/dying cells, they tend to acquire immunostimulatory capability [5–6]. DAMPs have the ability to activate immune cells like macrophages, certain T cells, NK cells, and dendritic cells (DCs) [7–8]. They also assist in opsonization and/or phagocytosis of cancer cells [9–10], activation of inflammasome and transcription of inflammatory gene programs in immune cells [11], and processing and presentation of proper tumor-associated antigens (TAA) [8]. Three major DAMPs, calreticulin (CRT), heat shock proteins 70 (HSP70), and high mobility group box 1 (HMGB1), have been identified [12]. The combination of these major DAMPs has been found to be associated with ICD induction [13].

Under physiological conditions, CRT is located in the endoplasmic reticulum (ER) and is involved in chaperone-related functions as well as alcium homeostasis and signaling [12]. The precursor of apoptosis is the translocation of CRT to the outer leaflet of the plasma membrane induced by therapy-mediated stress in the ER [14–15]. When CRT externalizes to the cell surface, they acts as an “eat me” signal for phagocytes, mostly DCs and macrophages, through their CD91 receptor [16]. Eventually, this leads to presentation of tumor antigens by antigen-presenting cells (APCs) and activation of T cells.

Owing to their widespread association with apoptotic cell death, heat shock proteins (HSPs) are called seasoned apoptotic DAMPs [6]. As highly conserved chaperones, HSPs be of great benefit to in structural folding of both newly synthesized as well as stress-modified protein [17]. Elevated expression of HSP70, either on the cell membrane or in extracellular space can be immunostimulatory. Photofrin-PDT could induce HSP70 translocation to the outer leaflet of plasma membrane of cancer cells [18]. Membrane (outer leaflet) association of HSPs has attracted special attention, because of their ability to activate innate immunity cells like DCs and NK cells [6]. Extracellular release of HSPs has been found to be capable of stimulating migration and maturation of DCs (upregulation of MHC class II, CD80, CD86, CD83, and CD40 molecules) as well as activation of NK cells [19].

Under normal conditions, HMGB1 protein acts as a DNA chaperone that modulates the transcriptional activity of various proteins, such as steroid hormone receptors, p53, and nuclear factor-κB (NF-κB) [20]. Moreover, HMGB1 facilitates recombination and participates in chromatin-level transcriptional regulation [20]. Interestingly, extracellular HMGB1 has been reported to be vital for the immunogenicity of ICD. CT26 murine colon adenocarcinoma cell line treated with chemotherapy and radiotherapy has been used as a prophylactic tumor vaccine [21]. However, it was observed that ability of mice to resist rechallenge was comprised when the mice were immunized with HMGB1-depleted CT26 cancer cells or by co-injection of HMGB1-specific antibody [21]. It has been reported for some time that large amount of HMGB1 as a DAMPs can be passively released by necrotic cells [6, 21]. In the context of ICD, HMGB1 stimulates the production of pro-inflammatory cytokines, such as TNF, IL-1, IL-6, and IL-8, from innate immune cells like neutrophils, monocytes, and macrophages [21].

Photodynamic therapy (PDT) is a multi-step procedure involving the selective uptake of a photosensitiser by the tumor tissue, followed by illumination of the neoplastic lesion with a light of appropriate wavelength able to trigger photochemical reactions that lead to tumor destruction [22]. Topical 5-aminolevulinic acid mediated PDT (ALA-PDT) is an established treatment for actinic keratosis, Bowen's Disease, superficial basal cell carcinoma, and other cancerous and precancerous skin diseases. PDT not only kills tumor cells directly but also rapidly recruits and activates immune cells favoring the development of antitumor adaptive immunity [23]. In recent reports [24–25], hypericin-based PDT (hyp-PDT) induced immunogenic apoptosis characterized by phenotypic maturation and functional stimulation of DCs as well as induction of a protective antitumor immune response [24].

In our recent study, we observed that ALA-PDT-treated tumor cells could induce maturations of DCs, including morphology maturation (enlargement of dendrites and increase of lysosomes), phenotypic maturation (MHC-II^high^, DC80^high^, and CD86^high^), and functional maturation (IFN-γ^high^ and IL-12^high^, and to induce T cell proliferation). Our results indicate that ALA-PDT can induce immunogenic apoptosis of tumor cells, and can be more effective in enhancing immunogenicity of tumor cells [26].

In this study, we investigated the effect of ALA-PDT induced DAMPs on immune cells. We focused on the stimulation of DCs by major DAMPs, enhanced the expression of calreticulin (CRT), heat shock proteins 70 (HSP70), and high mobility group box 1 (HMGB1), either individually or in combination.

## RESULTS

### Induction of SCC tumors in mice

**Figure 1:**
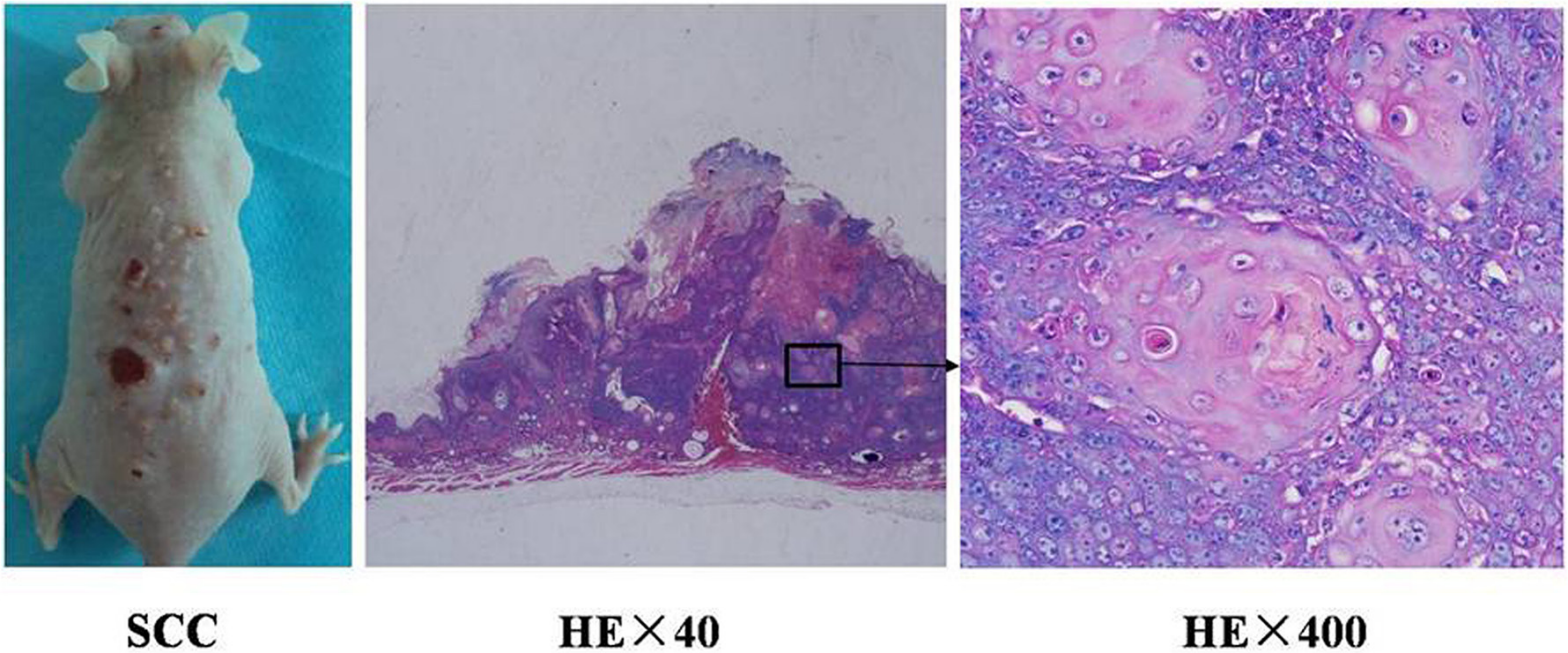
Histopathological analysis of UV-induced SCC tumors in the skin of mice **A.** SCC tumors with various sizes in mice. H&E stained SCC tumor tissue was observed with optical microscope at 40x **B.** and 400x **C.** The histological examination showed that a large number of atypical cells, pathological mitotic, and keratosis beads in tumor tissue.

Continued UV irradiation on SKH-1 mice lasted for 4 months until needle-size papules began to appear. Then SCC tumors continued to grow without irradiation. One month after stopping irradiation, multiple tumors became palpable and some tumors began to develop surface erosion and ulcers, as shown in Figure 1A. Hematoxylin/eosin stain was used to identify characteristic pathological changes of SCCs (Figure 1). Some neoplastic cells were in mitosis and others were dyskeratotic or necrotic, and a large number of atypical cells, pathological mitotic, and keratosis beads in tumor tissue were observed (Figure 1B and 1C).

### *In situ* immunogenic SCC cell death induced by ALA-PDT treatment

**Figure 2:**
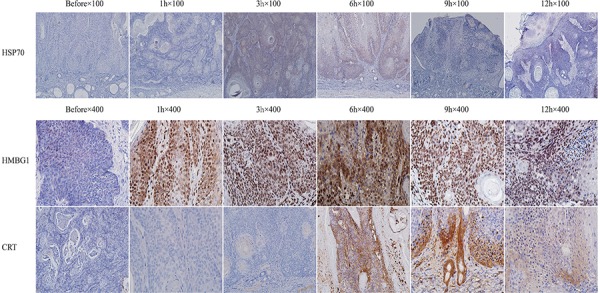
Expressions of HSP70, HMGB1, and CRT after ALA-PDT treatment in tumor tissue Tumor tissue was collected 1, 3, 6, 9, and 12 h after treatment, stained and observed under different magnifications: for HSP70 at 100× (upper panel) and for HMGB1 and CRT at 400× (middle and lower panels, respectively). The expressions of all three DAMPs were positively increased between 3 to 9 h after ALA-PDT, reaching their peak values at 6 h.

To investigate the induced antitumor immune responses, the UV-induced SCC tumors in mice were treated by ALA-PDT. Histological examination of tissue taken from treated tumor sites was performed 0 to 12 h after ALA-PDT. Untreated tumor tissue was used for comparison. Immunohistochemistry was employed to observe expression of CRT, HSP70, and HMGB1 in treated tumors. As shown in Figure 2, positive staining for HSP70 was observed 3 h and 6 h after ALA-PDT and noticeable reduction of HSP70 expression was seen 9 h after treatment. HMGB1 expression markedly increased 1 h after ALA-PDT (Figure 2), compared with untreated tumor tissue, and reached a peak at 6 h before beginning to decline. Similarly, CRT expression on tumor tissue increased considerably between 0 to 9 h after ALA-PDT (Figure 2), before declining. It is worth noting that the cells mainly underwent apoptosis, as observed in our previous studies [27].

### Expression of intracellular CRT, HSP70, and HMGB1 induced by ALA-PDT treatment

**Figure 3:**
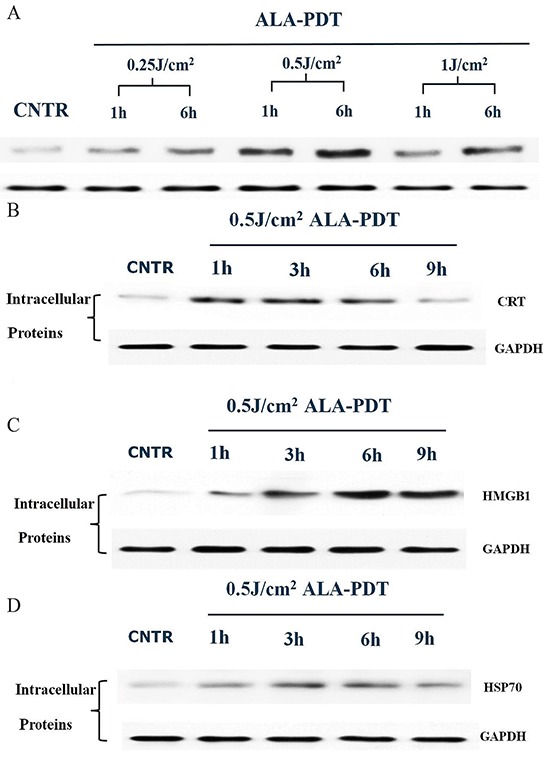
Intracellular expression of DAMPs in PECA cells after ALA-PDT treatment **A.** Expression of intracellular CRT. PECA cells were treated by ALA-PDT with different doses (0.5J/cm^2^, 1J/cm^2^, 2J/cm^2^), and CRT expression was analyzed by western blot at 1 h or 6 h after treatment. The highest expression of CRT was observed under the treatment with the light dose of 0.5J/cm^2^. Intracellular expressions of CRT **B.** HMGB1 **C.** and HSP70 **D.** in PECA cells at different time points (0 h to 9 h) after treatment with a light dose of 0.5J/cm^2^. The expressions of HSP70, HMGB1, and CRT reached their peak values between 3 to 6 h.

To determine ALA-PDT induced intracellular DAMPs, expressions of CRT, HSP70, and HMGB1 of PECA cells treated by ALA-PDT (0.25J/cm^2^, 0.5J/cm^2^, 1J/cm^2^) were analyzed by western blot analysis. As shown in Figure 3A, expression of CRT was the highest at 0.5J/cm^2^. At 0.5J/cm^2^, CRT expression markedly increased between 1 h to 6 h after treatment and noticeably decreased after 9 h (Figure 3B). HMGB1 expression increased 1 h after treatment, reached a peak at 6 h, and started decreasing at 9 h (Figure 3C). ALA-PDT increased HSP70 expression of PECA cells between 3 and 6 h after treatment, as shown in Figure 3D.

### Exposure of CRT and HSP70 on tumor cell surface induced by ALA-PDT

**Figure 4:**
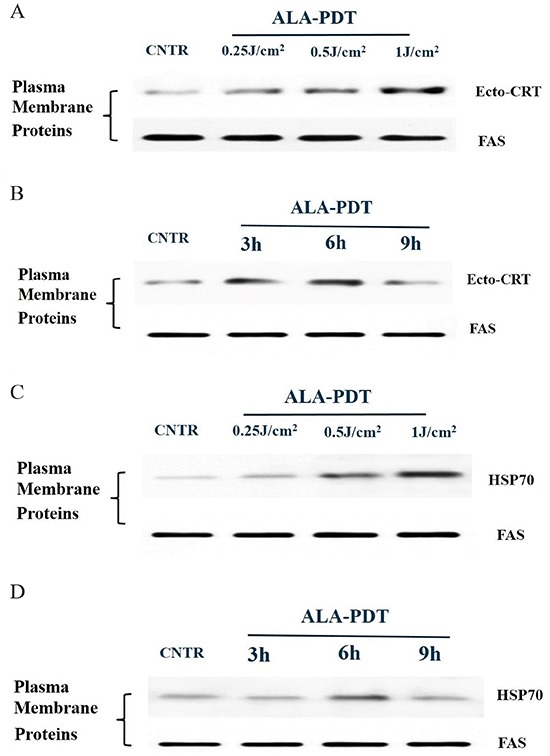
Membranal exposure of DAMPs on the surface of PDT-treated PECA cells PECA cells were treated with ALA-PDT and collected, the exposures of CRT and HSP70 on the surface of tumor cells were analyzed by western blot. Untreated cells were used for negative comparison. **A.** CRT exposure on PECA cells treated by ALA-PDT with different doses (0.5J/cm^2^, 1J/cm^2^, 2J/cm^2^). **B.** CRT exposure at different time points (3 h to 9 h after treatment) with a light dose of 0.5J/cm^2^. **C.** HSP70 exposure of PECA cells treated by ALA-PDT with different doses (0.5J/cm^2^, 1J/cm^2^, 2J/cm^2^). **D.** HSP70 exposure at different time points (3 h to 9 h after treatment) with a light dose of 0.5J/cm^2^. ALA-PDT induced surface exposures of HSP70 and CRT as a function of light dose, and the exposures reached the peak values 6 h after treatment and then began to decline.

HSP70 and CRT exposure on the surface of PECA cells was analyzed by western blot at different time points after ALA-PDT (0.25J/cm^2^, 0.5J/cm^2^, 1J/cm^2^). CRT and HSP70 expressions on surface of PECA cells increased as a function of light dose (Figure 4A, 4C). Exposures of CRT and HSP70 on the cell surface reached the peak values at 6 h after ALA-PDT before beginning to decline (Figure 4B, 4D).

### Secretion of HMGB1 and HSP70 induced by ALA-PDT treatment

**Figure 5:**
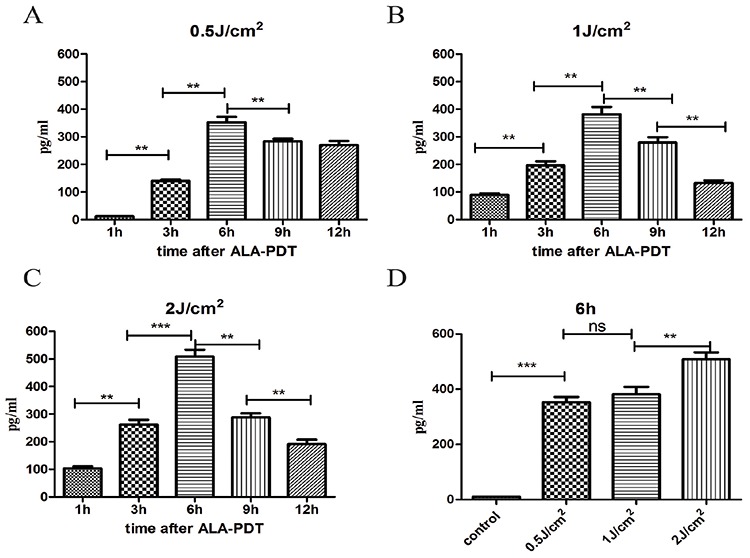
Extracellular release of HMGB1 induced by ALA-PDT PDT-stimulated release of HMGB1 from PECA cells at different time points (1 h to 12 h) at 0.5J/cm^2^ **A.** 1J/cm^2^ **B.** and 2J/cm^2^ **C.** using ELISA assay. **D.** The peak values of HMGB1 6 h after treatment. Statistical analysis was performed by *t*-test; *p < 0.05, **p < 0.01 and ***p < 0.001; ns = not statistically significant. Means ± SD are shown from 3 independent experiments.

**Figure 6:**
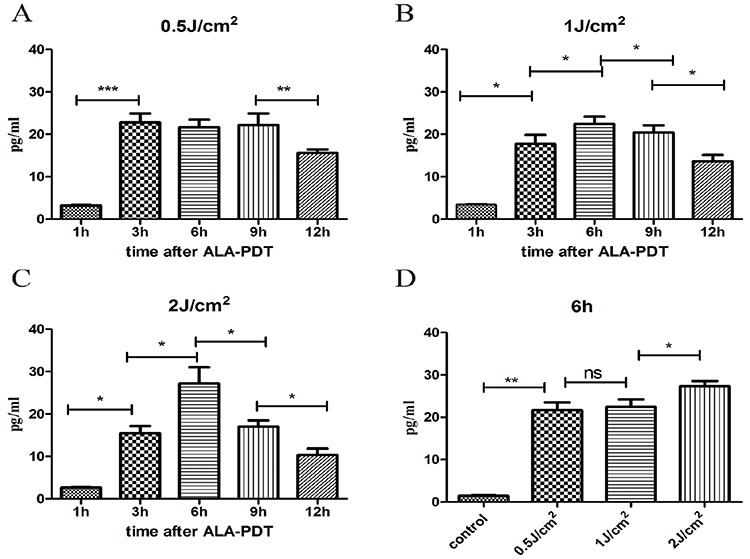
Extracellular release of HSP70 induced by ALA-PDT PDT-stimulated release of HSP70 from PECA cells at different time points (1 h to 12 h) at 0.5J/cm^2^ **A.** 1J/cm^2^ **B.** and 2J/cm^2^ **C.** using ELISA assay. **D.** The peak values of HSP70 6 h after treatment. Statistical analysis was performed by *t*-test; *p < 0.05, **p < 0.01 and ***p < 0.001; ns = not statistically significant. Means ± SD are shown (*n* = 5 from 3 independent experiments).

HMGB1 and HSP70 releases were measured by ELISA in the supernatants of PECA cell culture between 1 h to 12 h after ALA-PDT treatment (0.5J/cm^2^, 1J/cm^2^, 2J/cm^2^). As show in Figure 5A–5C, PECA cells began to release HMGB1 1 h after treatment and HMGB1 reached a peak value 6 h after treatment. As shown in Figure 5D, 6 h after treatment, ALA-PDT of all light doses induced significant release of HMGB1, although the difference between 0.5J/cm^2^ and 1J/cm^2^ was not significant. HSP70 secretion from PECA significantly increased 3 h after ALA-PDT treatment (0.5J/cm^2^), compared with the untreated cells, and significantly dropped at 12 h (*p* < 0.01) (Figure 6A). As show in Figure 6B, 6C, HSP70 reached a peak value 6 h after ALA-PDT treatment (1J/cm^2^, 2J/cm^2^). However, there is no significant difference in HSP70 release between cells treated at 0.5J/cm^2^ or 1J/cm^2^ (Figure 6D).

### Effect of DAMPs on phenotypic maturation of DCs

**Figure 7:**
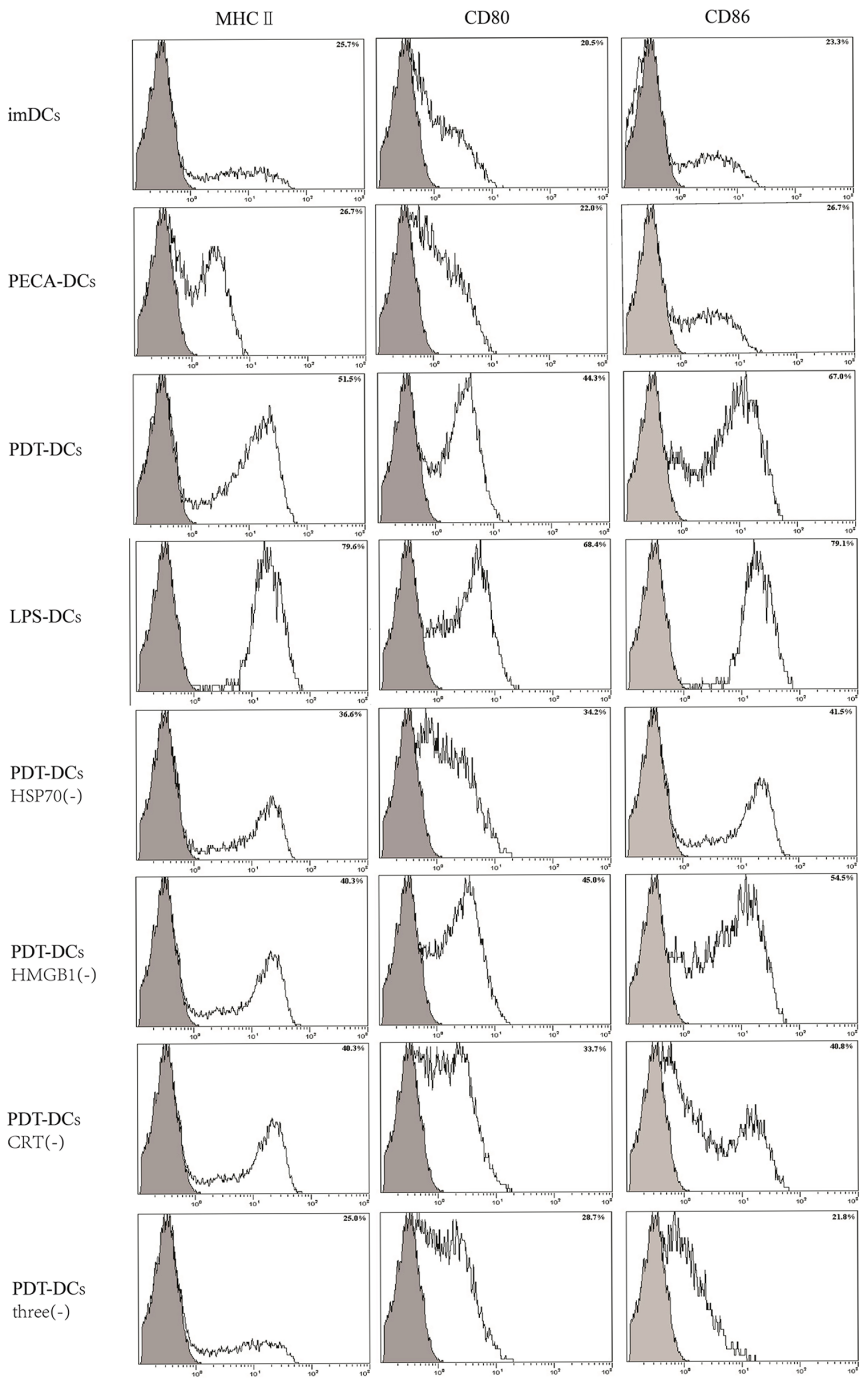
Expressions of MHC-II, CD80, and CD86 on the surface of DCs stimulated by treated PECA tumor cells PECA cells were collected 6 h after ALA-PDT treatment (0.5 mM ALA, 0.5 J/cm^2^), then co-incubated with imDCs for another 24 h. The stimulation of DCs was at the similar level as that of LPS (3^rd^ and 4^th^ panels). Blocking HSP70, HMGB1 and CRT individually reduced the expression of MHC-II, CD80, and CD86 on DCs (5, 6, and 7^th^ panels); however, blocking all three DAMPs resulted in the least DC activation (8^th^ panel). PECA-DCs represents untreated PECA cells were collected and incubated with imDCs for 24 h, and PDT-DCs represents DCs stimulated with PDT-treated PECA cells. Three represents HSP70, HMGB1 and CRT, and (−) represents blocking.

The role of DAMPs in phenotypic maturation of DCs simulated by PDT-treated PECA cells was determined by blocking CRT, HSP70, and HMGB1 during co-incubation of PDT-treated PECA cells and imDCs for 24 h. Neutralizing antibodies of HSP70, HMBG1, and CRT were used either individually or in combination. Untreated imDCs were used as negative control and imDCs stimulated by LPS were used as positive control. Flow cytometric analysis showed that the expressions of CD80, CD86, and MHC-II molecules on the surface of DCs stimulated by PDT-treated cells increased significantly compared with controls, as shown in Figure 7. When HSP70, HMGB1, and CRT were blocked individually, the activation of DCs was suppressed; however, blocking all three DAMPs resulted in the least DC activation (Figure 7).

### Effect of DAMPs on functional maturation of DCs

**Figure 8:**
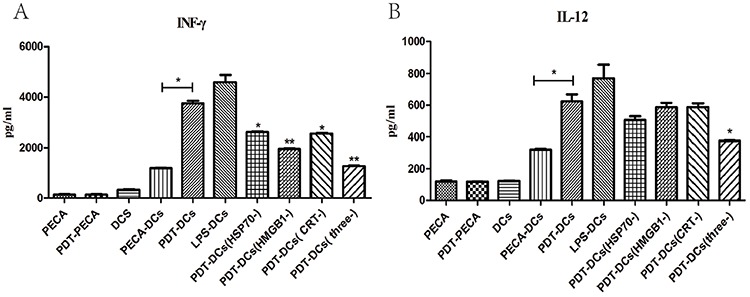
Secretion of IFN-γ and IL-12 from DCs under different stimulations Secretion of IFN-γ **A.** and IL-12 **B.** from DCs after incubation with ALA-PDT-treated PECA cells was quantified in cell culture supernatants using ELISA assay, with or without HSP70, HMGB1 or CRT neutralizing antibodies in the co-incubation medium. Unstimulated, immature DCs were used for negative control and DCs incubated with LPS were used for positive control. ALA-PDT-treated PECA cells induced the secretion of IFN-γ and IL-12 markedly higher than that induced by untreated PECA cells (*p* < 0.05). When HSP70, HMGB1 and CRT were blocked individually, or in combination, IFN-γ secreted from DCs was markedly reduced (*p* < 0.05). When all of three antibodies were included in the co-incubation medium, IL-12 secreted from DCs significantly decreased when compared with PDT-DCs (*p* < 0.05). Statistical analysis was performed by *t*-test; *p < 0.05 and **p < 0.01 with respect to the PDT-DCs (unless otherwise specified); ns = not statistically significant. Means ± SD are shown (*n* = 5 from 3 independent experiments.)

The functional state of DCs incubated with ALA-PDT-treated PECA cells was analyzed for the presence of IFN-γ and IL-12 using ELISA assay. As shown in Figure 8, ALA-PDT-the treated cells secreted IFN-γ and IL-12 at a level markedly higher than that by untreated cells (*p* < 0.05). When HSP70, HMGB1 and CRT were blocked individually, or in combination, IFN-γ secreted from DCs reduced significantly (*p* < 0.05). When blocking agents for CRT, HSP70, and HMGB1 were used individually, IL-12 dropped slightly; however, blocking all three DAMPs resulted in significant reduction of IL-12 (*p* < 0.05).

### Antitumor immunity in mice induced by ALA-PDT-treated SCC cells

**Figure 9:**
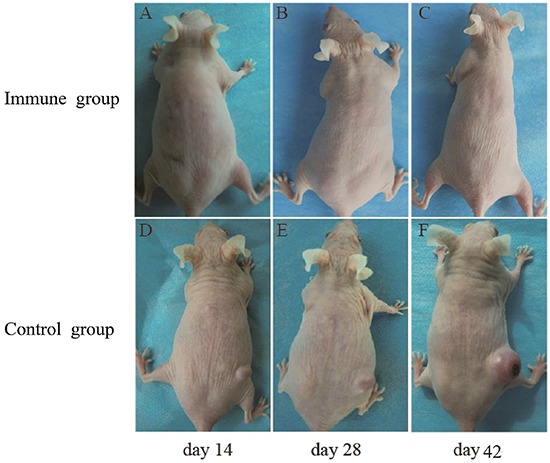

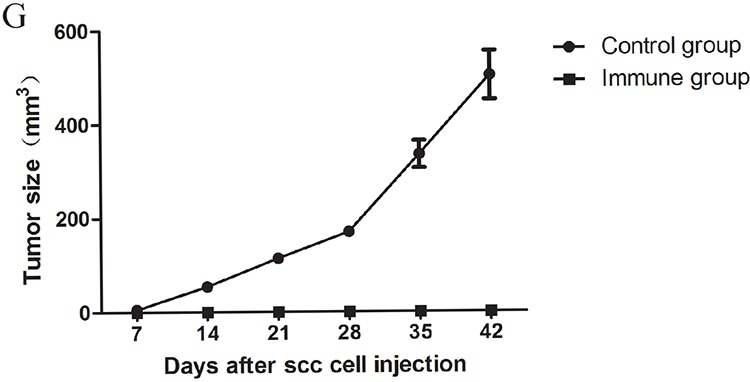
Tumor growth in SKH-1 mice after implantation of SCC tumor cells A group of naïve SKH-1 mice (*n* = 10) were immunized by SCC cells collected from tumor-bearing mice 6 h after ALA-PDT treatment (0.5 mM ALA, 0.5 J/cm^2^). The mice were immunized three times with 10-day intervals. Seven days after the third immunization, mice were challenged with viable SCC cells. Mice immunized with PDT-treated SCC cells showed no tumor growth **A–C.** whereas all mice pre-treated with PBS only developed tumors **D–F. G.** showed tumor growth curves of different groups.

We immunized naïve SKH-1 mice with ALA-PDT-treated SCC cells, which were collected from tumor-bearing SKH-1 mice. After the immunization was performed three times, the mice were challenged with viable SCC cells. Forty-two days after injection with viable SCC cells, no tumors were observed in the immunized mice, while all control mice experienced tumor growth (Figure 9).

## DISCUSSION

Cutaneous squamous cell carcinoma (SCC) is a common human skin cancer in elderly patients and its incidence has been on the rise due to the increasing life expectancy [28]. The lesions are often located on the head, the face, and the vulva, and the external genitalia. For SCCs of large size or at vulnerable sites, traditional treatments, such as surgery, chemotherapy, cryotherapy, and radiotherapy, were unsatisfactory. Such treatments have certain disadvantages, including destruction of normal tissue structure and function, dose limitation, and high rate of recurrence [29].

Poorly immunogenic tumor cells often lead to metastasis and recurrence. To effectively treat poorly immunogenic cancers, stimulating the immune system, particularly enhancing the immunogenicity of the tumor cells, is believed to be a winning strategy.

Photodynamic therapy (PDT) is effective for the treatment of cutaneous cancers [30–32]. Our recent study indicated that tumor cells treated by ALA-DPT stimulated phenotypic maturation (MHCII^high^, CD80^high^, CD86^high^) and functional stimulation (INF-γ^high^, IL-12^high^, IL-10^low^ and to induce T cell proliferation) of DCs [26]. In this study, we further investigated the effect of ALA-PDT on inducing DAMPs and stimulating DCs, leading to tumor-specific immune responses.

We established ultraviolet induced SCC in mice (Figure 1). Our previous study, we identify the death of SCC tumor through apoptosis or necrosis pathway using TUNEL staining [27]. We observed apoptosis cells start to reach a peak at 6 hours after ALA-PDT. Using the tumor tissue from the tumor-bearing mice after ALA-PDT treatment, we observed increased expressions of DAMPs induced by ALA-PDT, as shown in Figure 2. Therefore, our results clearly demonstrated the capability of PDT in inducing DAMPs *in vivo*.

The induction of DAMPs by ALA-PDT was further supported by our findings in *in vitro* study. While the primary skin tumor model induced by UV in SKH-1 mice was a general cutaneous SCC animal model, similar to the primary SCC tumors in human, PECA tumor cells is a cutaneous SCC cell line of mice, which have the biological characteristics of SCC. Therefore, we used PECA tumor cells to study the mechanism of PDT induced DAMPs. As shown in Figure 3, expressions of intracellular CRT, HMGB1, and HSP70 of PECA cells increased after ALA-PDT, depending on the light dose. Furthermore, we investigated exposures of CRT and HSP70 on the surface of ALA-PDT-treated PECA cells. Exposure of CRT increased as a function of the light dose, and reached a peak value 6 h after ALA-PDT (Figure 4A, 4B). Exposure of CRT on surface of dying cancer cells is critical for ICD [33]. In a recent report, the externally added recombinant CRT boosted antitumor response elicited by PDT or PDT-generated vaccines [34].

Our data revealed that ALA-PDT was capable of exposing HSP70 on the plasma membrane of tumor cells increased with light dose; the exposure of HSP70 was the highest 6 h after ALA-PDT treatment (Figure 4C, 4D). Membranal HSPs can stimulate DCs and NK cells to produce pro-inflammatory cytokines and process/react to tumor antigens [6]. As seasoned apoptotic DAMPs, HSP70 could also be secreted from PDT-treated tumor cells. As shown in Figure 6, ALA-PDT of all light doses induced significant release of HSP70 6 h after treatment. Extracellular release of HSPs could be complexed to tumor-associated peptides or antigens. Then these HSP-peptide complexes could be taken up by APCs, and presented to and activate T lymphocytes, initiating adaptive immune response [35].

We also demonstrated that ALA-PDT induced extracellular release of HMGB1 in a dose dependent manner; the expression of HMGB1 reached a peak value 6 h after ALA-PDT (Figure 5). Extracellular HMGB1 activates macrophages and DCs, and recruits neutrophil though a range of receptors, including TLR2, TLR4, and RAGE [36–38]. HMGB1 as DAMPs has been widely demonstrated in necrotic cancer cells [21]. Recently, apoptotic cancer cells were also shown to release HMGB1 at some points in the irrespective execution phases [39].

Significant increase in expression of all three DAMPs of PECA cells was observed after the treatment by ALA-PDT at 0.5J/cm^2^ as compared with untreated tumor cells. It has been proposed that apoptotic tumor cells can induce more effective antitumor immune responses than necrotic tumor cells [40–41]. Our previous study showed that ALA-PDT at 0.5J/cm^2^ induced maximum number of apoptotic cells, while ALA-PDT at 2J/cm^2^ resulted in most necrotic cells [26]. Moreover, expression of the three DAMPs reached a peak value 6 h after ALA-PDT. Thus, we used the dose of 0.5J/cm^2^ and post-treatment period of 6 h as the optimal parameters of ALA-PDT for most of our experiments in the present study.

DCs are the major link between the innate and adaptive immune responses. DCs can exhibit various states and perform different functions [42]. Tumor cells undergoing ICD can promote maturation of DCs. These mature DCs are capable of presenting antigen to and priming the adaptive immune cells. The distinction between immature and mature DCs lies in the changes in their phenotypic level and functional level [42].

In the present study, tumor cells treated by ALA-DPT stimulated phenotypic maturation (MHCII^high^, CD80^high^, CD86^high^). When HSP70, HMGB1, and CRT were blocked individually, expressions of CD80, CD86, and MHC-II of DCs were negatively affected, while blocking all three DAMPs resulted in the least DC activation (Figure 7). Our results suggest that HSP70, HMGB1, and CRT, particularly in combination, are important to stimulate phenotypic maturation of DCs by ALA-PDT-treated tumor cells.

PDT-treated tumor cells also induced functional stimulation (INF-γ^high^, IL-12^high^) of DCs as shown in Figure 8A. When blocking agents for CRT, HSP70, and HMGB1 were used individually, IL-12 dropped slightly; however, blocking all three DAMPs resulted in significant reduction of IL-12 (*p* < 0.05), as shown in Figure 8B. IFN-γ produced by mature DCs contributed to Th1 polarization [43]. Th1-type cells were critical for the optimal generation and durability of specific cytotoxic T lymphocytes (CTLs) that promote antitumor immunity [44–45]. Owing to the activation of CD8+T cells and the inhibition of angiogenesis, IL-12 has been reported consistently to have potent antitumor activity [46]. Our data demonstrate that HSP70, HMGB1, and CRT significantly decrease the induction of IFN-γ and IL-12 in DCs (Figure 8), hence being crucial for DCs maturation and antitumor immune response.

In this study, the antitumor immunity of the ALA-PDT-treated SCC cells was tested in SKH-1 mice. As shown in Figure 9, ALA-PDT-treated SCC cells worked as “tumor vaccines” in preventing SCC growth in immunized mice. Protection against tumor growth at the challenge site was a sign of anti-tumor immunity, established by the activation of DCs through the induced DAMPs.

In conclusion, this study show that ALA-PDT generated ICD, which triggers exposure of CRT and HSP70 on the cell membrane, as well as releases of HSP70 and HMGB1 to extracellular space. Our results also support that DMAPs play a pivotal role in ALA-PDT induced phenotypic maturation and functional maturation of DCs. Furthermore, ALA-PDT-treated SCC cells provided protection against cutaneous squamous cell carcinoma in mice. Our findings indicate that ALA-PDT is capable of inducing DAMPs and enhancing immunogenicity of SCC cells.

## MATERIALS AND METHODS

### Animals and cell lines

Male SKH-1 hairless mice (6–8 weeks old, Jackson Laboratories), free of skin injuries, were used. Cutaneous squamous cell carcinoma (SCC) on the back of mice was induced by solar-simulated ultraviolet irradiation (Solar UV Simulator, SIGMA Shanghai, China) as described previously [29]. SCC tumors were excised from mice sacrificed and then enzymatically disaggregated into single cell suspensions for experiments. SCC cells were then washed in PBS and maintained in DMEM medium supplemented with 10% fetal bovine serum (FBS) (Gibco, Billings, MT, USA), 2 mM L-glutamine (Gibco) and 1% penicillin/streptomycin antibiotics (Gibco) at 37°C in an atmosphere of 5% CO_2_ [47–48].

SCC cell line PECA (CLS Cell Lines Service GmbH, Eppelheim, German) was also used in this study. The cells were maintained in RPMI 1640 medium supplemented with 10% fetal bovine serum (FBS) (Gibco, Billings, MT, USA), 2 mM L-glutamine (Gibco) and 1% penicillin/streptomycin antibiotics (Hyclone) at 37°C in an atmosphere of 5% CO_2_.

### Chemicals and reagents

RPMI 1640 cell culture medium, PBS, and penicillin/streptomycin were obtained from Hyclone (Thermo Scientific, Waltham, Massachusetts, USA). Fetal bovine serum (FBS) was obtained from Gibco (Billings, MT, USA). ALA hydrochloride powder was from Shanghai Fudan-Zhangjiang Bio-Pharmaceutical Co, Ltd (Shanghai, China). Rabbit polyclonal anti-HSP70 and mouse monoclonal anti-HMGB1 (Cell Signaling Technology, USA), rabbit polyclonal anti-calreticulin (abcam, USA), mouse monoclonal anti-GAPDH and mouse monoclonal anti-FAS (Santa Cruz, USA) were used. In addition, we used mouse HSP70 ELISA Kit (ENEO, USA), mouse HMGB1 ELISA Kit (SHINO-TEST, Japan), mouse IFN-γ and IL-12 ELISA Kit (Boster, China), rabbit anti-Mouse CD80-PE, rabbit anti-Mouse CD86-PE, rabbit anti-Mouse MHC-II-PE, and rat IgG2a K Isotype Control PE (Biolegend, USA). Neutralizing antibodies for CRT, HSP70, and HMGB1 were purchased commercially (Santa Cruz, USA).

### PDT treatment

For *in vitro* study, SCC cells were incubated with ALA (0.5 mM) in full serum-free medium for 5 h at 37°C, then washed twice with PBS and exposed to the light dose of 0.5 J/cm^2^ (632.8 nm, 10 mW/cm^2^). For *in vivo* study, 8% ALA cream was topically applied onto tumor surface for 3 h. Mice were irradiated by a helium-neon laser (632.8 nm) at a power density of 100 mW/cm^2^ and energy density of 15 J/cm^2^.

### Immunohistochemical studies

At designated time points (0 to 12 h) after treatment, mice were sacrificed. Freshly isolated tissue was stored in formalin and 5 μm sections were de-waxed (30 min 56°C, 2 × 10 min xylene), followed by rehydration, antigen unmasking, and blocking. Then the samples were stained with anti-HSP70, anti-HMGB1, and anti-CRT primary antibodies at 1 μg/ml in blocking solution for 30 min at room temperature. Slides were rinsed in PBS and incubated with goat anti-rabbit IgG secondary antibody (Boster, China) diluted in blocking solution for 30 min. Slides were inculcated with strept avidin-biotin complex (Boster, China) for 30 min, rinsed in PBS, and stained using a DAB chromagen and hematoxylin counterstain, and observed under a light microscope. PBS was used for negative control sections.

### Western blot

To determine ALA-PDT induced HSP70, HMGB1, and CRT expression, membrane proteins of ALA-PDT-treated PECA tumor cells were extracted with a membrane protein Extraction Kit (BOSTER, China). PECA cells were lysed with radioimmunoprecipitation assay (RIPA) buffer (Tris base 50 mM, NaCl 150 mM, NP40 1%, sodium deoxycholate 0.25%, EDTA 1 mM) containing a protease inhibitor cocktail (Roche Diagnostics, Mannheim, Germany). The protein concentration was measured using a BCA protein assay kit (Pierce, Rockford, IL, USA). Equal amounts of proteins were separated by SDS-polyacrylamidegel electrophoresis (PAGE) on 10% gels and transferred to nitrocellulose membranes (Bio-Rad Laboratories, Hercules, CA, USA). The membranes were blocked with 5% (w/v) non-fat milk in TBS [Tris-buf-fered saline (pH 7.4)] containing 0.1% (v/v) Tween 20 (TBST) and then incubated with primary antibodies (anti-HSP70, anti-HMGB1 and anti-CRT antibody) overnight at 4°C. The membranes were then washed three times (15 min each) with TBST, and incubated with an alkaline phosphatase-conjugated secondary antibody (1:2000) for 1 h at room temperature. The color reaction was developed using NBT (p-nitro-blue tetrazolium chloride) and BCIP (5-bromo-4-chloro-3-indolyl-phosphate; Sigma).

### Quantification of HMGB1 and HSP70 release

In order to evaluate release of HMGB1 in response to ALA-PDT, PECA cells were cultured in six well tissue culture plates, and treated with ALA-PDT with different fluences (0.5J/cm^2^, 1J/cm^2^, 2J/cm^2^). After centrifugation, the supernatants were collected and analyzed using an ELISA-based HMGB1 and HSP70 detection kits according to the manufacturer's instructions, at determined time points (0 to 12 h) after PDT treatment.

### Flow cytometric analysis of DCs

Six hours after treatment, PECA cells without treatment or treated by ALA-PDT (0.5 mM ALA, 0.5J/cm^2^) were incubated with immature dendritic cells (imDCs), at a ratio of 1:20 (imDCs:PECA) for 24 h. As block agents, neutralizing antibodies of HSP70, HMBG1, and CRT were used either individually, or in combination, during the co-incubation with imDCs and PECA cells. Unstimulated imDCs were used for negative control and LPS-treated DCs (4μg/ml LPS for 24 h) were used for positive control. The DCs were collected and then washed in PBS, followed by incubation with the following antibodies: anti-mouse CD80-PE, anti-mouse CD86-PE, and anti-mouse MHC-II-PE for 40 min at 4°C in the dark. After incubation, the cells were washed in PBS and analyzed with a FACS flow cytometer (Becton Dickinson, Mountain View, CA).

### Detection of cytokines from DCs

To detect cytokines (IFN-γ, IL-12) secreted from DCs, imDCs were incubated with treated and untreated PECA cells at a ratio of 1:20 (imDCs:PECA) in 24-well tissue culture plates. Blocking agents for CRT, HSP70, and HMGB1 were used either individually, or in combination, during co-incubation. After 24 h, the supernatants were collected and divided into different groups. The cytokines (IFN-γ, IL-12) were determined by ELISA assay according to manufacturer's instructions. Immature DCs were used for negative control and DCs incubated with 4μg/ml LPS for 24 h were used for positive control.

### Detection of ALA-PDT-treated SCC cells induced immune response

SCC cells were treated by ALA-PDT (0.5 mM ALA, 0.5 J/cm^2^). Six hours after treatment, cells were collected, washed twice in PBS, and resuspended in PBS (for injection). To detect immune response induced by ALA-PDT-treated SCC cells, female SKH-1 mice, age 6–8 weeks were randomly divided into two groups (10 per group). The mice were immunized by injecting subcutaneously in the left flank with ALA-PDT treated tumor cells (6 × 10^6^ cells in 0.2 ml solution). Immunization was performed three times with a 10-day interval. Mice in control group were injected with 0.2 ml PBS. Mice were then challenged with 1 × 10^6^ viable SCC cells in the right flank 7 days after the third immunization. Following the challenge, the mice were monitored every day.

### Statistical analyses

Data are presented as mean ± standard deviation (unless other-wise specified). Data were analyzed with GraphPad Prism 5 software. Statistical analyses were performed using *t*-test and *P* < 0.05 was considered statistically significant.
